# T-Cell Receptor Rearrangements in Early Stages of Mycosis Fungoides May Be Associated with Pronounced Copy Number Variations: A Prognostic Factor?

**DOI:** 10.3390/cancers17030556

**Published:** 2025-02-06

**Authors:** Carsten Hain, Cassandra Cieslak, Jörn Kalinowski, Rudolf Stadler

**Affiliations:** 1 Center for Biotechnology (CeBiTec), Bielefeld University, 32429 Bielefeld, Germany; chain@cebitec.uni-bielefeld.de (C.H.); joern@cebitec.uni-bielefeld.de (J.K.); 2 Department of Dermatology, Johannes Wesling Medical Centre, University Hospital of the Ruhr-University of Bochum (UKRUB), University of Bochum, 32429 Minden, Germany; cassandra.cieslak@ruhr-uni-bochum.de

**Keywords:** CTCL, T-cell receptor, diagnostic, genomic aberrations, sequencing

## Abstract

Mycosis fungoides (MF), the most common form of cutaneous T-cell lymphoma, poses significant diagnostic challenges due to its overlap with benign inflammatory skin diseases and the absence of specific symptoms. Accurate early diagnosis and stratification of patients by progression risk are essential for effective treatment. This study proposes a proof-of-concept for integrating T-cell receptor (TCR) clonality analysis with somatic mutation profiling to enhance diagnostic confidence and prognostic accuracy in early-stage MF. This study’s methodology comprised the analysis of nine patients with early MF (stages IA/IB) using whole-exome sequencing and TCR repertoire profiling. The analysis revealed the presence of clonal TCR rearrangements in seven patients, while somatic mutations were identified in two. A notable finding was a recurrent chromosome 7 trisomy in these two cases. The patients were stratified into three molecular profiles: (1) somatic mutations with clonal TCR rearrangement (n = 2), (2) clonal TCR rearrangement without somatic mutations (n = 4), and (3) neither somatic mutations nor clonal TCR rearrangement (n = 3). These findings emphasise the heterogeneity of MF and underscore the limitations of relying solely on TCR clonality or mutation burden for diagnosis. This study underscores the potential of somatic mutations as diagnostic markers to distinguish MF from benign conditions and as prognostic indicators for disease progression. A combined genetic approach may refine treatment decisions, particularly for patients with higher tumor cell fractions and pronounced genetic alterations. Despite the limited size of the cohort, the results advocate for larger, multi-center studies to validate these findings and integrate genetic analyses into routine MF management.

## Simple Summary

This study aims to improve the diagnosis and prognosis of MF by combining two genetic analysis methods: T-cell receptor (TCR) clonality analysis and somatic mutation profiling. This study explores improving MF diagnosis and prognosis by combining T-cell receptor (TCR) clonality analysis and somatic mutation profiling. Key findings include the detection of TCR clonal rearrangements in seven of nine early-stage patients and somatic mutations in two patients. This allowed for the classification of the patients into three molecular profiles. Potential benefits might be enhanced diagnostic accuracy, better prognostic insights, and refined treatment decisions. However, the small sample size limits conclusions. Future research should focus on larger, multi-center studies to validate the results and integrate genetic analyses into MF management.

## 1. Introduction

Mycosis fungoides (MF), the most common form of cutaneous T-cell lymphoma, presents with limited skin lesions and shows highly heterogenous disease progression between patients. Due to often non-specific skin lesions and certain overlap to benign inflammatory skin diseases, MF is a diagnostic challenge, and the current diagnostic procedure combines clinical, histological, immunohistological, and, increasingly, molecular data. Despite this, there is a pronounced diagnostic delay in MF and sometimes the non-specific symptoms are attributed to other diseases such as eczema or parapsoriasis [1,2]. In addition to a confident and timely diagnosis, the identification and differentiation of indolent patients and patients with a high risk of progression are desirable to administer treatment accordingly. The main molecular feature used for diagnosis is the detection of a clonal T-cell receptor (TCR) rearrangement pattern. Exact quantification of the malignant TCR rearrangement is also an accurate prognostic marker. A frequency of the malignant clone in the total T-cell population above 25% indicates an elevated risk of progression into late-stage MF [3]. However, benign inflammatory diseases can also lead to a clonal TCR rearrangement, making a distinction difficult between those and MF purely based on TCR clonality analysis [4]. Therefore, a combination of TCR clonality analysis and the detection of further somatic mutations might be beneficial for a confident diagnosis and an accurate prognosis.

## 2. Materials and Methods

### 2.1. Sample Collection

Punch biopsies from 9 patients with MF were obtained with written consent. Patient characteristics are shown in Table 1. Fresh punch biopsies were stored in MACS^®^ Tissue Storage Solution (Miltenyi Biotec, Bergisch Gladbach, Germany). In eight of nine cases, sampling took place shortly after initial diagnosis. In one case (patient 1), the diagnosis of MF had already been established for nine years

### 2.2. Tissue Processing and Whole-Exome Data Generation

The workflow from freshly obtained punch biopsies to somatic variant calls was carried out as described previously [5]. In brief, cells were dissociated and enriched for CD3+ cells and DNA was isolated. A normal control DNA from patient-matched blood was used. From this DNA, whole-exome sequencing libraries were constructed, sequenced, and analyzed from SNVs and CNVs using GATK workflows.

### 2.3. T-Cell Receptor Sequencing

DNA from CD3+ cells from the punch biopsies was subjected to T-cell receptor profiling using the next-generation sequencing protocol from the Euroclonality consortia with minor modifications [6]. Data analysis was carried out with MixCR [7]. Tumor clone frequency (TCF) was calculated based on the two most frequent alleles for T-cell receptor gamma or the single most frequent allele for T-cell receptor beta.

## 3. Results

**Figure 1:**
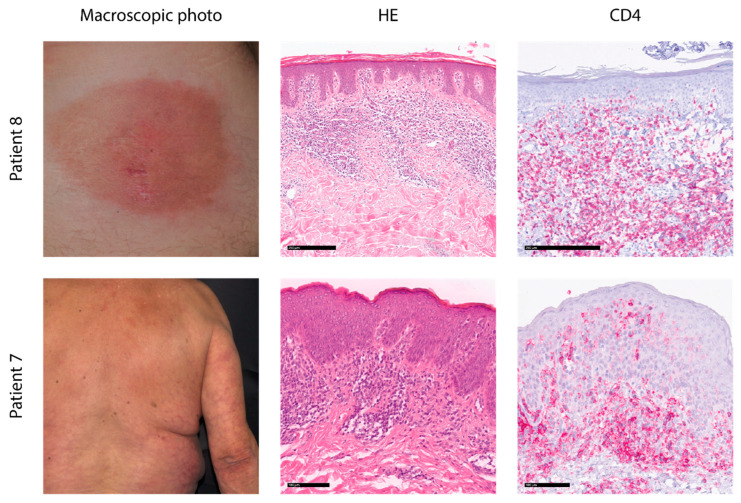
Histological and macroscopic pictures of exemplary patients. Patient 8 presents with a well-circumscribed patch at the lower right back. Histology shows subepidermal bandlike, slightly epidermotropic lymphocytes with a CD4 phenotype. Patient 7 shows disseminated patches on the upper back and arm with a subepidermal dense epidermotropic infiltrate of small lymphocytes with a CD4 phenotype. HE: hematoxylin–eosin staining.

This study aims to provide a proof-of-concept for the combination of different genetic assays in the context of confident diagnosis and prognosis of early-stage MF. To achieve this, we measured somatic mutations and TCR rearrangement frequencies in a skin biopsy using current standard methods like whole-exome sequencing and T-cell receptor sequencing, respectively. The patient cohort consisted of nine patients who were referred to the University Department of Dermatology in Minden after non-response to standard eczematous treatment. The clinical diagnosis of early MF was made based on the clinical picture of each patient with an asymmetric, singular manifestation, the corresponding histological and immunohistological profiles of epidermotropic T cells, and hyperpigmented, mainly CD4-positive T cells, without major spongiosis in the epidermis. Exemplary data for two patients are shown in Figure 1. All cases showed limited MF (stages IA or IB), and while eight cases were early MF, shortly after diagnosis and without prior therapy, one case was an early-stage MF with a seven-year history of the disease. The patients were treated after the diagnosis of early MF according to the EORTC guideline with skin-directed therapies, e.g., chlormethine gel., PUVA, or peg. interferon in combination with Bexarotene, and one case after progression during follow-up to stage IB received low-dose radiotherapy (see Table S1).

**Figure 2:**
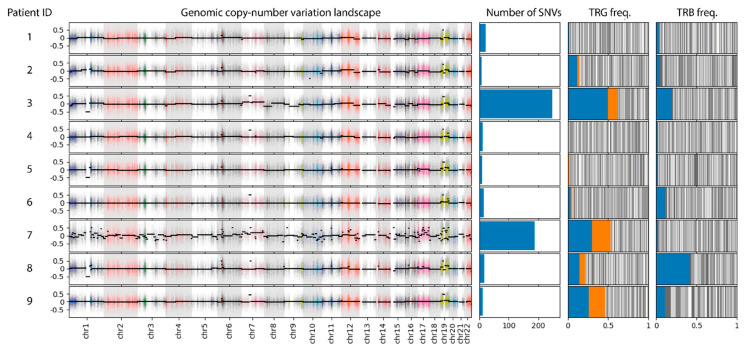
Genetic data of nine early-stage MF patients. For each patient, the copy number landscape, the number of SNVs, and the distribution of T-cell receptor alleles for the gamma (TRG) and beta (TRB) gene is shown. Positive values in the CNV plot indicate amplification, while negative values indicate deletions. Clear CNVs are only visible in samples 3 and 7, best indicated by the chr7 amplification present in both samples. This correlates with the number of SNVs, which is above potential background noise in samples 3 and 7. The T-cell receptor repertoire is depicted as a stacked bar of individual allele frequencies (grey bars). The alleles used for TCF calculation are marked in blue and orange (TRG) and blue (TRB).

For all patients in the cohort, we performed enrichment of CD3+ cells from fresh tumor biopsies. The enriched cells as well as patient-matched normal samples were subjected to whole-exome sequencing as described earlier to detect somatic single-nucleotide variants (SNVs) and copy number variants (CNVs) [5]. Concurrently, the T-cell receptor gamma and beta repertoire and tumor clone frequency (TCF) were assessed in the CD3+ cells using DNA-based amplicon sequencing using a modified Euroclonality workflow [6,8]. The TCF calculation took into account the most frequent (TCR beta chain) or the two most frequent clones (TCR gamma chain). Extensive SNVs and CNVs were detected in two of the nine patients (patients 3 and 7, Figure 2). Of note is the chromosome 7 trisomy present in both samples. The remaining patients showed no confident SNVs or CNVs beyond technical noise. TCR sequencing detected clonal rearrangements for the TCR gamma chain in five patients (patients 2, 3, 7, 8, and 9) and for the TCR beta chain in five patients (patients 2, 3, 6, 8, and 9). With the exception of patients 6 and 7, patients were simultaneously clonal for the gamma and beta TCR chains. Aggregation of somatic variation and TCR rearrangement data separated individual patients into three groups: samples with somatic mutations and a clonal TCR rearrangement (patients 3 and 7) as well as samples without somatic mutations (patients 1, 4, and 5) or with TCR clonality (patients 2, 6, 8, and 9).

## 4. Discussion

We performed a combined analysis of somatic mutations and TCR rearrangement in a cohort of early-stage MF patients, which stratified the patients into categories based on the presence and absence of somatic mutations and a clonal TCR rearrangement. This dual readout of genetic changes has several advantages. Somatic mutations, especially in the extensiveness observed here in samples 3 and 7, are a clear argument in favor of MF and thus distinguish it from benign inflammatory diseases. In this respect, the somatic mutational burden should be included in the diagnosis of MF. Prospectively, the exact nature of individual mutations might also influence the diagnostic algorithm of MF.

Of special note is the chr7 trisomy found in samples 3 and 7. This alteration is recurrent in various cancers [9,10,11] and abundant in CTCL, [12,13,14] has biological consequences like altered expression and nuclear organization [15], and might hint to a general mis-segregation defect in the malignant cells of these cells. With a progressive understanding of the functional consequences of individual mutations, it may be possible to establish a correlation between genetic changes and potential symptoms and the course of the disease.

For the cohort presented here, the performed analysis provides only a snapshot of the current genetic landscape. In this sense, a further examination of the molecular changes, i.e., an analysis of the TCR rearrangements and the somatic mutations during follow-up in the next few years, is particularly interesting. The major question is if the individual groups (somatic mutations yes/no and clonal TCR rearrangement yes/no) develop differently. The cohort used here is too small to answer this question comprehensively and should therefore be understood as a starting point for a future combined analysis of genetic changes in early-stage MF in a multi-center approach. Higher case numbers using such an approach would allow definitive statements to be made which, due to the small cohort size in this study, cannot be made here.

However, it seems that patients with a higher fraction of tumor cells and more pronounced or easy-to-detect somatic mutations require combination therapies and detectable and extensive genetic changes might call for a more active therapy than the current wait-and-see approach for early and limited MF as well as a closer follow-up. In any case, this speculative connection should be observed in further cases. Going forward, a more comprehensive integration of genetic analysis in the diagnosis and prognosis as well as choice of therapy of MF is a necessary aim.

In addition, this study again highlights the technical challenges that accompany the genetic analysis of early-stage MF, namely, the high heterogenity and deceptively low fraction of malignant cells. Striking is that the TCR analysis shows a high tumor clone frequency even in samples that do not show somatic mutations, although the detection limit for SNVs of 5% is well below the measured values for TCR clonality, e.g., in cases 2, 8, and 9 [16]. An incomplete enrichment of the CD3+ cells as well as amplification bias during TCR quantification might explain this discrepancy.

To tackle this issue and reveal the underlying mutational processes in the earliest stages of MF, a more sophisticated preparation of individual cell populations in the tumor biopsy is needed. Additionally the application of single-cell techniques like single-cell RNA [17] or ATAC sequencing [18] to a large cohort of early-stage MF patients might reveal the basic genomic changes in this elusive disease. Generally, progress in the understanding of the fundament mechanisms in MF genetics will help to develop better diagnostic and prognostic avenues.

## 5. Conclusions

In conclusion, this study provides valuable insights into the genetic landscape of early-stage mycosis fungoides (MF) and highlights the potential benefits of combining T-cell receptor (TCR) clonality analysis with somatic mutation profiling. The findings underscore the heterogeneity of MF and reveal three distinct molecular profiles among patients:

(1) those with both somatic mutations and clonal TCR rearrangement;
(2) those with TCR clonality but without detectable somatic mutations;
(3) those without either somatic mutations or clonal TCR rearrangement.

The detection of extensive somatic mutations, particularly the recurrent chromosome 7 trisomy, in some patients offers a clear distinction between MF and benign inflammatory conditions [1]. This suggests that incorporating the somatic mutational burden into the diagnostic algorithm for MF could improve diagnostic accuracy.

Furthermore, this study emphasizes the technical challenges in analyzing early-stage MF, such as the low fraction of malignant cells and the discrepancy between TCR clonality and detectable somatic mutations. These findings call for more sophisticated cell preparation techniques and the application of single-cell technologies to better understand the fundamental genomic changes in MF.

While the small cohort size limits definitive conclusions, this research serves as a proof-of-concept and a starting point for future larger-scale, multi-center studies. The integration of a comprehensive genetic analysis in MF diagnosis, prognosis, and treatment selection appears to be a promising avenue for improving patient care.

Ultimately, this study paves the way for a more nuanced approach to MF management, potentially allowing for more personalized treatment strategies based on individual genetic profiles. As our understanding of the molecular mechanisms underlying MF continues to grow, it is likely to lead to better diagnostic tools and more effective therapeutic interventions for patients with this challenging condition.

## Figures and Tables

**Table 1:** Clinical and molecular data of 9 MF patients.

ID	Sex	Age	Stage/TNMB	Number SNVs	TCF		Therapy	Response
					*TRG*	*TRB*		
1	M	74	IA/T1bN0M0B0	21	2.5	2.9	UVB 311 nm, Bexaroten, Chlormethin gel 0.1%	CR
2	F	63	IA/T1aN0M0B0	8	14.8	4.5	UVB 311 nm, Mometasonfuroat 0.1%	PR
3	M	74	IB/T2bN0M0B0	248	61.6	19.1	Peg. Interferon 135 µg, PUVA, low-dose radiotherapy	PR
4	M	72	IA/T2bN0M0B1	11	1.0	1.2	UVB 311 nm, Peg. Interferon 135 µg	CR
5	M	61	IIA/T2N1M0B0	9	2.1	1.5	UVB 311 nm, Bexaroten	CR
6	M	64	IA/T1aN0M0B0	15	5.6	11.1	UVB 311 nm	PR
7	M	63	IA/T1aN0M0B0	188	53.7	1.8	Chlormethin gel 0.1%	PR
8	M	60	IA/T1aN0M0B0	16	21.9	41.8	Chlormethin gel 0.1%, PUVA, Clobetasol salve	PR
9	M	68	IA/T1aN0M0B0	11	46.4	10.6	UVB 311 nm	PR

Table legend: Single-nucleotide variants (SNVs); tumor clone frequency (TCF) as determined by T-cell receptor gamma (TRG) or T-cell receptor beta (TRB); patient response is either complete response (CR) or partial response (PR). Additional information is in Table S1.

## Data Availability

Data are available from the authors upon request.

## Supplementary Materials

The following supporting information can be downloaded at https://www.mdpi.com/article/10.3390/cancers17030556/s1, Table S1: Patient Characteristics.
