# Supplementary material for: T-Cell Receptor Rearrangements in Early Stages of Mycosis Fungoides May Be Associated with Pronounced Copy Number Variations: A Prognostic Factor?
Source: Cancers (Basel). 2025 Feb 6;17(3):556. doi: 10.3390/cancers17030556 (PMC11817690; doi:10.3390/cancers17030556)
Supplement: Supplementary file 1 [file cancers-17-00556-s001.zip › cancers-3357408-supplementary.pdf]

Supplementary Materials: T-Cell Receptor Rearrangements in Early Stages of Mycosis Fungoides May Be Associated with Pronounced Copy Number Variations: A Prognostic Factor?

Table S1. Patient Characteristics. For each patient information such as sex, age, date of initial diagnosis, MF classification and staging, administered therapy and subsequent response as well as genetic data like number of SNVs and clonality of T-cell receptor gamma (TRG) and beta (TRB) are shown. T-cell receptor clonality (TCF) is a numerical representation of the degree of clonality of the T-cell receptor repertoire.

Patient ID	Sex	Age	Initial diagnosis	Tumor classification	Stage	Therapy	Response	Number of SNVs	TCF TRG	TCF TRB	Clonality (NGS)
1	M	74	04/2013	T1bN0M0B0	IA	UVB 311 nm, Bexaroten, Chlormethin gel 0.1 %	CR	21	2.3	2.4	polyclonal
2	F	63	03/2021	T1aN0M0B0	IA	UVB 311 nm, Mometasonfuroat 0,1%	PR	8	18.9	3.8	clonal TRG
3	M	74	07/2022	T2bN0M0B0	IB	Peg. Interferon 135 µg, PUVA, low-dose radiotherapy	PR	248	55.4	13.9	clonal
4	M	72	02/2022	T2bN0M0B1	IA	UVB 311 nm, Peg. Interferon 135 µg	CR	11	1	1.3	polyclonal
5	M	61	07/2022	T2N1M0B0	IIA	UVB 311 nm, Bexaroten	CR	9	2.1	1.5	polyclonal
6	M	64	07/2022	T1aN0M0B0	IA	UVB 311 nm	PR	15	5	10	clonal
7	M	63	07/2022	T1aN0M0B0	IA	Chlormethin gel 0.1 %	PR	188	45.4	2	clonal TRG
8	M	60	11/2021	T1aN0M0B0	IA	Chlormethin gel 0.1 %, PUVA, Clobetasol salve	PR	16	18.7	39.4	clonal
9	M	68	08/2022	T1aN0M0B0	IA	UVB 311 nm	PR	11	41.3	9	clonal TRG

Table legend: Single-nucleotide variants (SNVs); tumor clone frequency (TCF) as determined by T-cell receptor gamma (TRG) or T-cell receptor beta (TRB); patient response is either complete response (CR) or partial response (PR).
